# Hepatoptosis with Gaseous Intestinal Cysts

**Published:** 1918-05

**Authors:** 


**Hepatoptosis With Gaseous Intestinal Cysts.** Laurent Moreau, Arch. d'Electricite Med., Sept., 1917, reports a case in a man aged 35, non-syphilitic, of unexplained origin, in which the diagnosis was made largely by radiology. The stomach was vertical and extended almost to the pubes. The shadow of the liver was distinct and above it, beneath the arch of the diaphragm, was a clear space, evidently indicating gaseous distension. The case was considered hepatoptosis by interposition, i.e., loops of intestine or some similar interposed body causing the liver to descend. Beclere had reported such a case due to a subphrenic abscess, in which gas had developed and Aubourg had reported an analogous case, not of hepatoptosis but of the interposition of a loop of colon in a fossa on the upper posterior surface of the liver. (Note: physical examination not very rarely suggests the interposition of intestine between the anterior abdominal wall and the antero-superior surface of the liver, though many such cases are undoubtedly deceptive). In the present case, the gaseous filling above the liver was due to gaseous intestinal cysts which are very rare, only about 50 cases in all having been reported in the literature, and whose pathogeny is not well understood. Turnure and Mauclaire have each reported a case quite in analogy with the present one. So far as known, these are the only three cases of this particular type of hepatoptosis by interposition thus far reported.

